# Effect of chewing gum on orthodontic pain in patients receiving fixed orthodontic treatment: a systematic review and meta-analysis

**DOI:** 10.1186/s40001-023-01467-y

**Published:** 2023-11-08

**Authors:** Qiushuang Guo, Chengcheng Liao, Xiaoyan Guan, Linlin Xiao, Meiling Xiang, Sicen Long, Jianguo Liu, Mingli Xiang

**Affiliations:** 1 Department of Orthodontics II, Affiliated Stomatological Hospital of Zunyi Medical University, Zunyi, 563000 China; 2 Oral Disease Research Key Laboratory of Guizhou Tertiary Institution, School of Stomatology, Zunyi Medical University, Zunyi, 563006 China

**Keywords:** Chewing gum, Orthodontic pain, Fixed orthodontic appliances, Meta-analysis

## Abstract

**Objectives:**

The objective of this systematic review and meta-analysis was to evaluate the effect of chewing gum on orthodontic pain and to determine the rate of bracket breakage associated with fixed orthodontic appliances.

**Methods:**

This review and its reporting were performed according to the Cochrane Handbook for Systematic Reviews of Interventions and the PRISMA guidelines. Six electronic databases were searched up to March 16, 2023, to identify relevant studies that met the inclusion and exclusion criteria. Furthermore, grey literature resources were searched. The Cochrane Collaboration Risk of Bias tool 2 was used to assess the quality of the included studies. Meta-analysis was conducted using RevMan, and sensitivity analysis and publication bias analysis were performed using STATA software. GRADE tool was used to evaluate the certainty of evidence.

**Results:**

Fifteen studies with 2116 participants were ultimately included in this review, and 14 studies were included in the meta-analysis. Compared with the blank group, chewing gum had a significant pain relieving effect at all times after fixation of the initial archwire (*P* ≤ 0.05). No significant difference was found between the chewing gum group and the analgesics group at any timepoints (*P* > 0.05). Only four studies evaluated the rate of bracket breakage and revealed that chewing gum did not increase the rate of bracket breakage. The sensitivity analysis showed that there was no significant difference in the pooled outcomes after the included studies were removed one at times, and Egger analysis revealed no significant publication bias in included studies (*P* > 0.05).

**Conclusions:**

Chewing gum is a non-invasive, low-cost and convenient method that has a significant effect on relieving orthodontic pain and has no effect on the rate of bracket breakage. Therefore, chewing gum can be recommended as a suitable substitute for analgesics to reduce orthodontic pain.

## Introduction

Orthodontic treatment guides tooth movement and bone remodeling to align dentition, coordinate jawbone, improve occlusal function, and enhance facial beauty by applying force through orthodontic devices. In recent years, the demand for orthodontic treatment has increased dramatically. However, orthodontic appliances and their generated orthodontic force often cause pain, anxiety and irritability, thereby having a negative impact on oral hygiene and patients’ daily lives [1]. Pain has become the main negative complication during orthodontic treatment, especially fixed orthodontic treatment [2]. Approximately 90–95% of patients report some level of discomfort or pain after applying fixed appliances, and they also feel pain after monthly return visits during long-term treatment [3]. Pain not only leads to low patient satisfaction and low compliance with orthodontic treatment, but also causes a large proportion of patients to discontinue or resist orthodontic treatment. Therefore, alleviating orthodontic pain is an urgent problem for orthodontists and patients.

Many pharmacological and nonpharmacological methods have been proposed to relieve orthodontic pain. Analgesics, mainly nonsteroidal anti-inflammatory drugs (NSAIDs), have obvious effects on reducing orthodontic pain [4]. However, analgesics have some side effects, such as gastric ulcers, gastric bleeding, thrombocytopenia, renal insufficiency, hepatotoxicity, atherosclerosis and hypertension [5, 6]. Moreover, some NSAIDs may hinder orthodontic tooth movement, which is detrimental to orthodontic treatment [7]. Therefore, most orthodontists do not prescribe analgesics to relieve orthodontic pain in clinical practice.

Nonpharmacological methods, such as low-level laser therapy (LLLT) [8–10], transcutaneous electrical nerve stimulation (TENS) [11, 12], low-intensity pulsed ultrasound (LIPUS) [13] and vibrating stimulation [14], plastic wafers [15, 16] and chewing gum [17], have emerged as approaches for relieving pain among patients treated with orthodontic appliances [18]. Among these nonpharmacological methods, chewing gum is a non-invasive, effective, convenient and inexpensive way to relieve orthodontic pain [19–22]. Many studies have confirmed that chewing gum has the same effect as analgesics for pain relief after fixing the initial arch wire [23–27]. In addition, M. Waheed-Ul-Hamid et al. found that chewing gum has a better pain relief effect than analgesics [28]. However, many reports have suggested that chewing gum has no clinically significant effect on orthodontic pain [29–31]. In addition, many orthodontists believe that chewing gum does not relieve orthodontic pain and does increase the rate of bracket breakages [32]. Therefore, it is still unclear whether chewing gum can relieve orthodontic pain and increase the rate of bracket loss; this lack of clarity is not conducive to the widespread use and promotion of chewing gum for orthodontic pain relief.

Former systematic reviews and meta-analyses have been conducted on the same topic with a very low quality of evidence: Jabr et al.'s study only included limited early phase studies and only evaluated pain value between chewing gum and conventional analgesic drugs [33]; Mando et al.'s study only evaluated pain score at its peak intensity [34]; these two studies assessed the risk of bias of these included studies according to the Cochrane Collaboration Risk of Bias tool 1 (RoB 1), and both studies included the experiments, where patients were treated with separators only, which may cause clinical heterogeneity, because various orthodontic appliances may lead to different force magnitudes and tissue response, thereby causing varying degrees of self-reported orthodontic pain. In addition, the previous studies did not include all relevant studies. Therefore, it is necessary to conduct a more scientific and accurate systematic review and meta-analysis.

Therefore, this review aimed to evaluate the effect of chewing gum on orthodontic pain and the rate of bracket breakage in patients who are planning to be treated with fixed orthodontic appliances. The finding can provide evidence-based recommendations for the clinical application and promotion of chewing gum to relieve orthodontic pain.

## Methods

This review was conducted in accordance with the Cochrane Handbook for Systematic Reviews of Interventions [35] and reported in line with the Preferred Reporting Items for Systematic reviews and Meta-analyses (PRISMA) [36]. In addition, the protocol of the present systematic review was registered in PROSPERO (#CRD42022360679).

### Search strategy

The Medline (via PubMed), Science Direct, Cochrane Library, Web of Science, WangFang and ZhiWang databases were searched until March 16th, 2023, to identify relevant articles. There were no publication language restrictions. In addition, the reference lists of relevant studies, including previously published reviews, were screened for additional studies. Unpublished articles were found by searching ClinicalTrials.gov and National Research Register. Grey literature resources were also searched using Open Grey, Google Scholar, and PROQUEST Thesis and Dissertations. The main search terms include “chewing gum”, “orthodontic” and “pain”. Two authors (Mingli Xiang and Qiushuang Guo), respectively, searched and selected the included studies according to the PRISMA method. We first excluded the studies by reviewing the titles and abstracts, and then, we selected the included studies by evaluating the full texts according to the eligibility criteria. Any differences between the authors were resolved through discussion with the third author (Xiaoyan Guan). Final decisions were taken after consensus had been reached.

### Eligibility criteria

The eligibility criteria were defined according to PICOS criteria (patient; intervention; comparison; outcome; study design).

Patient: participants were treated with fixed orthodontic appliances.

Intervention: chewing gum after fixation of the initial archwire.

Comparison: blank group: no intervention or taking placebo after initial archwire fixation; Analgesics group: taking analgesics after fixation of the initial archwire.

Outcome: primary outcome, pain score assessed by visual–analogue scale (VAS) or numeric rating scale (NRS); Secondary outcome: rate of bracket breakage.

Study design: randomized controlled trials (RCTs).

### Data collection

Study characteristics data were extracted: (1) author's name and publication year, (2) setting, (3) participants' characteristics, (4) bracket and archwire, (5) groups, (6) outcomes and (7) conclusion. orthodontic pain usually begins at 2 h after initial archwire placement, peaks at 24 h, and lasts for 7 days [37]. Therefore, this study evaluated patients' pain scores (mean and standard deviation) at 2 h, 6 h, 12 h, 24 h, 2 d, 3 d and 7 d after initial archwire fixation. Pain scores can be quantified using the VAS scale (10 cm or 100 mm) and the Numeric Rating Scale (10 cm). To standardize the pain scales to a single scale, we assumed that 10 cm VAS and 10 cm NRS were equivalent, and these scales were converted to 100 mm VAS by multiplying the pain scores by 10 [38]. If orthodontic pain was recorded in different occlusal states (e.g., resting, biting, etc.) in one study, we combined these pain values to obtain a single estimate according to previous studies [39, 40]. The rate of bracket breakage can also be evaluated and synthetized if there is sufficient data in these included studies. When these data were reported only graphically, it could also be extracted using the Windows-based digitizing computer program UnGraph (version 5.0; Biosoft, Cambridge, United Kingdom) [41]. If these data are not available directly from the articles, they can be calculated [42, 43] and obtained by contacting the corresponding author for the numerical data.

### Quality assessment

Two authors (Mingli Xiang and Qiushuang Guo) independently assessed the Risk of Bias of these included studies according to the Cochrane risk-of-bias tool for randomized trials (RoB 2) [44]. This includes the following domains: (D1) randomization process, (D2) deviations from intended interventions, (D3) missing outcome data, (D4) measurement of the outcome, and (D5) selection of the reported result. The studies were rated as having a low risk of bias, some concerns of bias, or high risk of bias for each. When a single study reported multiple outcomes of interest, the overall risk of bias was assessed rather than the risk of bias for each outcome.

The strength of the body of evidence was assessed using the Grading of Recommendations Assessment, Development and Evaluation (GRADE) tool [45]. This tool evaluates the quality of evidence in the following domains: study design, risk of bias, inconsistency, indirectness and imprecision.

### Statistical analysis

The primary outcome was patient-reported pain scores at 2 h, 6 h, 12 h, 24 h, 2 d, 3 d or 7 d after initial archwire fixation. The mean differences and standard error were combined using RevMan 5.1 (Cochrane Collaboration, Copenhagen, Denmark) [46]. Data were considered suitable for pooling if the retrieved studies met to the selected criteria. The statistical significance of the hypothesis test was set at *P* < 0.05 (two-tailed *z* tests). We chose a random effects model to estimate all pooled data considering the inherent differences in these studies. Heterogeneity was assessed using the I^2^ index. I^2^ index ≥ 50% indicates moderate heterogeneity and I^2^ index ≥ 75% indicates high heterogeneity. If high heterogeneity existed, sensitivity analyses were performed using the ‘metaninf’ command in Stata 17.0 (StataCorp, College Station, TX) [47] to evaluate the effect of individual studies on the overall mean difference. The Egger’s rank correlation test was conducted to detect publication bias if the number of included studies exceeded 8.

## Results

### Searching and selection results

**Fig. 1:**
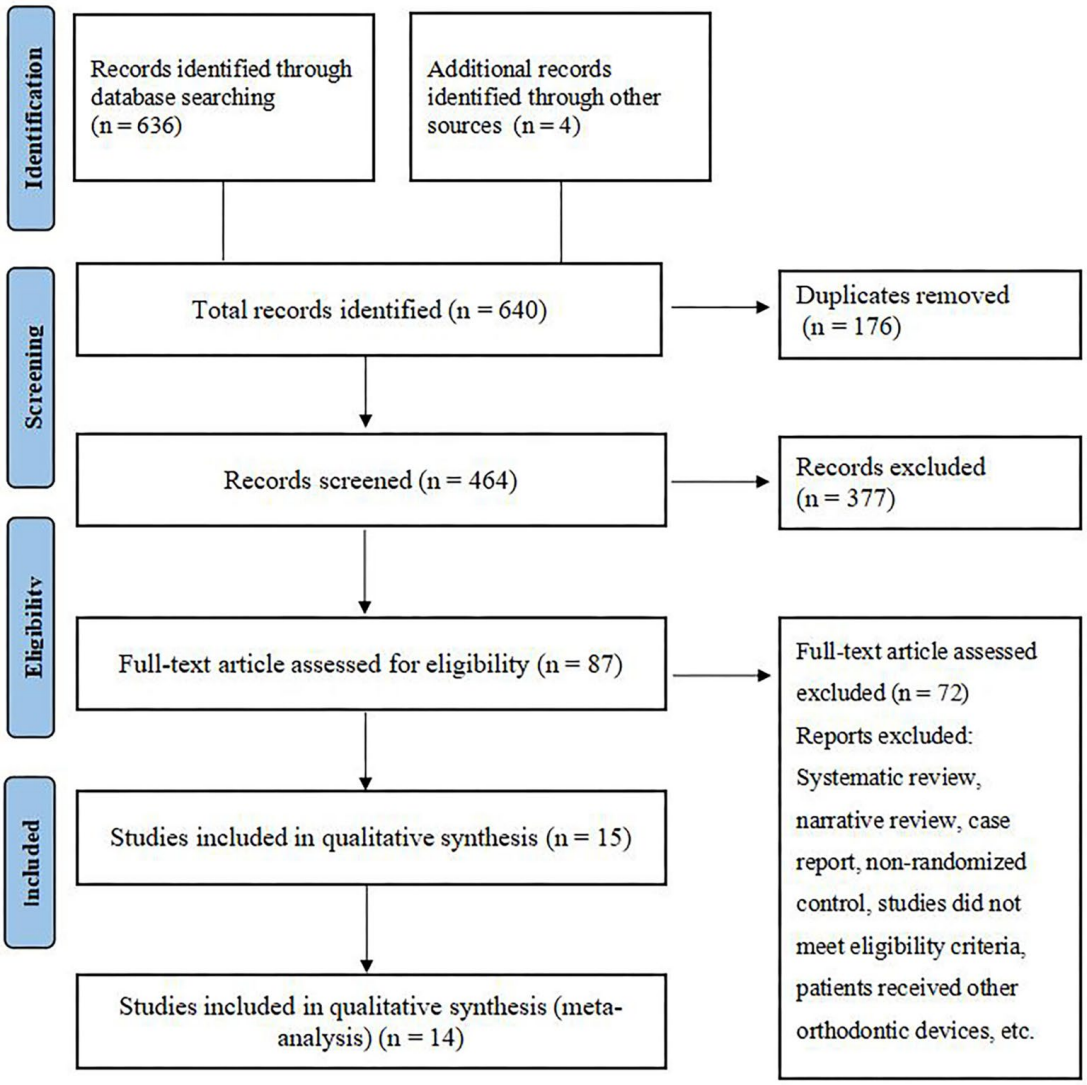
Flowchart of studies identification

A total of 640 studies were identified from the search strategy, 3 of which were derived from the reference lists of relevant studies and 1 from ClinicalTrials.gov. After removing duplicates, 464 remained; after screening the title and abstract, 377 were excluded; and 87 were excluded after evaluating the full text according to the eligibility criteria. Finally, 15 studies [19–31, 48, 49] were included in qualitative synthesis, and 14 studies [19–31, 49] were included in meta-analysis. The PRISMA flow diagram is shown in Fig. 1.

### Characteristics of the included studies

The characteristics of the included studies are summarized in Table 1. All included studies were RCTs, and 2116 patients were included. Patients received fixed orthodontic appliances with the initial aligning archwire only and without other auxiliary orthodontic devices, such as transpalatal arch, headgear, mini-screw, etc. These patients were excluded when they had some conditions affecting their pain scores, such as oral ulcers, oral diseases, taking analgesics recently, etc. In addition, tooth extraction for orthodontic treatment was conducted at least 2 weeks before the experiment [22, 24, 26, 28]. Six studies [19, 21, 29–31, 49] evaluated the effect of chewing gum on pain control compared to the blank group, five studies [23, 26–28, 48] analysed pain scores comparing the chewing gum group with analgesics, and four studies [20, 22, 24, 25] assessed the pain value in the chewing gum group, analgesics group, and blank group. Patients in eight studies [19, 21–24, 26, 31, 48] received fixed orthodontic treatment on bimaxillary arch, patients in five studies [20, 27–30] received only one arch, and two studies [25, 49] did not specify two or one arch. The aligning archwire included 0.012" NT [19, 21], 0.014" NT [20, 22, 29–31, 49] and 0.016" NT [23, 24, 26, 28], and the archwire size was unclear in 3 studies [25, 27, 48]. Fourteen studies quantified pain values using the VAS scale, and one study applied the NRS scale [22]. Two studies [20, 27] recorded pain scores when resting and biting, one study [48] recorded pain scores when biting and chewing, one study [26] recorded pain scores when fitting posterior teeth, biting and chewing, two studies [22, 24] recorded pain scores when chewing, biting, fitting anterior teeth and fitting posterior teeth, and other studies did not specify the occlusal state when pain was recorded. Only four studies evaluated the rate of appliance breakage [23, 27, 48, 49] and found that chewing gum did not increase the rate of bracket breakage when compared to the control group or analgesics group.

**Table 1:** Summary of the data from the included studies

Author, year	Setting	Sample size (n) and age (y)	Participants' characteristics	Bracket and Archwire	Groups	Outcomes	Conclusion
Celebi et al. 2022	Turkey	57, 12–24	3–6 mm maxillary crowding, no planned extraction, fixing appliances only in the upper arch	0.018 × 0.025" Roth prescription brackets (American Orthodontics, Sheboygan, Wis, USA), 0.014" NT archwire (TP Orthodontics, La Porte, Ind, USA)	CG group (19) Blank group (19) Mechanical vibration group (19)	Pain score: 10 cm VAS	Chewing gum has no clinically significant pain relief effect on orthodontic pain
Basam et al. 2022	India	42, 18–25	4–9 mm crowding, tooth extracted, fixing appliances in both arches	M.B.T brackets (3 M UnitekTM Gemini Metal Brackets, USA), 0.016" NT archwire	CG group (21) Analgesics group (21)	Pain score: 10 cm VAS	Chewing gum was not inferior to pre-emptive tenoxicam for pain control
Santos et al. 2021	Brazil	106, ≧12	mild-to-moderate dental crowding, fixing appliances only in the upper arch	0.022 × 0.028″ brackets, 0.014 NT archwire (Morelli, Sorocaba-SP, Brazil)	CG group (26) Analgesics group (53) Blank group (27)	Pain score: 100 mm VAS	Chewing gum may be a nonpharmacological alternative for orthodontic pain relief at 2 and 3 days after initial archwire placement
Celebi et al. 2021	Turkey	63, 12–24	3–6 mm crowding, no planned extraction, fixing appliances only in the upper arch	0.018 × 0.025″ Roth prescription brackets and tubes, 0.014" NT archwire	CG group (21) Blank group (21) Laser group (21)	Pain score: 10 cm VAS	Chewing gum had no clinically significant effect on orthodontic pain
Delavarian et al. 2020	Kerman	66, 12–30	4–8 mm crowding, extraction of two maxillary and two mandibular premolars, fixing appliances in both arches	0.022 × 0.028″ MBT brackets (Ortho Organizers, USA), 0.014″ NT initial archwires (G&H, USA)	CG group (22) Blank group (22) Analgesics group (22)	Pain score: 10 cm NRS	Chewing gum has no effect on bracket breakage and is beneficial for pain relief during orthodontic treatment
Shayea et al. 2020	Saudi Arabia	105, 15–35	1–4 mm crowding, no planned extraction, fixing appliances in both arches	0.016″ NT archwires	CG group (35) Analgesic group (35) Bite wafer group(35)	Pain score: 10 cm VAS; Bracket breakage	Chewing gum has the same pain relief effect as ibuprofen for orthodontic pain and has no clinically or statistically significant effect on bracket detachment
Alqareer et al. 2019	Kuwait	75, 12–31	fixing appliances in both arches	0.022″ MBT and 0.014" archwires	CG group (38) Blank group (37)	Pain scores: 100 mm VAS; Patients’ overall subjective assessment of pain; Analgesics use	Chewing gum three times a day does not appear to significantly reduce orthodontic pain compared to placebo
Alshammari et al. 2019	Saudi Arabia, Sweden	60, 12–18	fixing appliances in one arch	0.012″ and 0.014″ round active TruFlex NT archwire (Ortho Technology) and 0.016 supercable archwire (SPEED supercable™	CG group (29) Analgesics group (31)	Pain score: 10 cm VAS; Bracket breakage	The effect of chewing gum and paracetamol on initial orthodontic pain relief appears to be equivalent. Short-term use of chewing gum is not a risk factor for bracket loss
Elvina et al. 2018	Indonesia	40, 18–40	NA	NA	CG group(10) Analgesic group (10) Blank group (10) Green tea group (10)	Pain score: 100 mm VAS	There was no significant difference between chewing gum and acetaminophen in the amount of pain reduction experienced after fixed orthodontic appliance placement
Ireland et al. 2016	England	1000, 11–17	undergoing fixed maxillary and mandibular appliance therapy	NA	CG group (503) Analgesics group (497)	Pain score: 10 cm VAS; Bracket breakage; Analgesics use	Chewing gum may reduce ibuprofen use for orthodontic pain but has no clinically or statistically significant effect on bond failure
W-U-H et al. 2016	Pakistan	250, 12–16	Severe/moderate crowding requiring first premolar extractions	Straight wire edgewise appliance system with 0.016" NT archwire (3 M Unitek)	CG group (125) Analgesics group (125)	Pain score: 10 cm VAS	Chewing gum showed more reduction in pain scores for orthodontic patients than ibuprofen
Liu et al. 2015	China	89, NA	Mild–moderate crowding, fixing appliances in both arches	Straight-Wire Appliance (Tomy), 0.012″ NT archwire	CG group (44) Blank group (45)	Pain score: 10 cm VAS; personality traits: EPQ	Chewing gum can significantly reduce orthodontic pain
Yang et al. 2013	China	140, > 10	Fixing appliances in both arches	0.012" NT archwire (Amondi LTD)	CG group(70) Blank group (70)	Pain score: 10 cm VAS; Personality traits: EPQ	Chewing gum can reduce pain during orthodontic treatment, especially for patients with an extroverted personality and a stable mind
Farzanegan et al. 2012	Iran	50, 13–18	4–8 mm crowding, extracting 4 first premolar, and fixing appliances in both arches	Standard edgewise system (0.018’’) and 0.016" NT archwire	CG group (10) Blank group (10) Analgesics group (10) Viscoelastic groups (10)	Pain score: 10 cm VAS	Chewing gum is effective for pain reduction in orthodontic patients and can be recommended as a suitable substitute to ibuprofen
Benson et al. 2012	UK	68, 11–18	Fixing orthodontic appliance in at least one dental arch,	Preadjusted edgewise appliances (0.022-inch slot, MBT prescription, Victory; 3 M, St Paul, MN, USA), 0.014" NT archwire	CG group (37) Blank group (31)	Pain score: 100 mm VAS; Analgesics use; Bracket breakage	Chewing gum significantly reduced pain from the fixed appliances and did not increase the incidence of appliance breakages

*CG* chewing gum; *VAS* visual–analog scale; *NRS* numeric rating scale; *EPQ* Eysenck personality questionnaire

### Risk of bias assessment

**Fig. 2:**
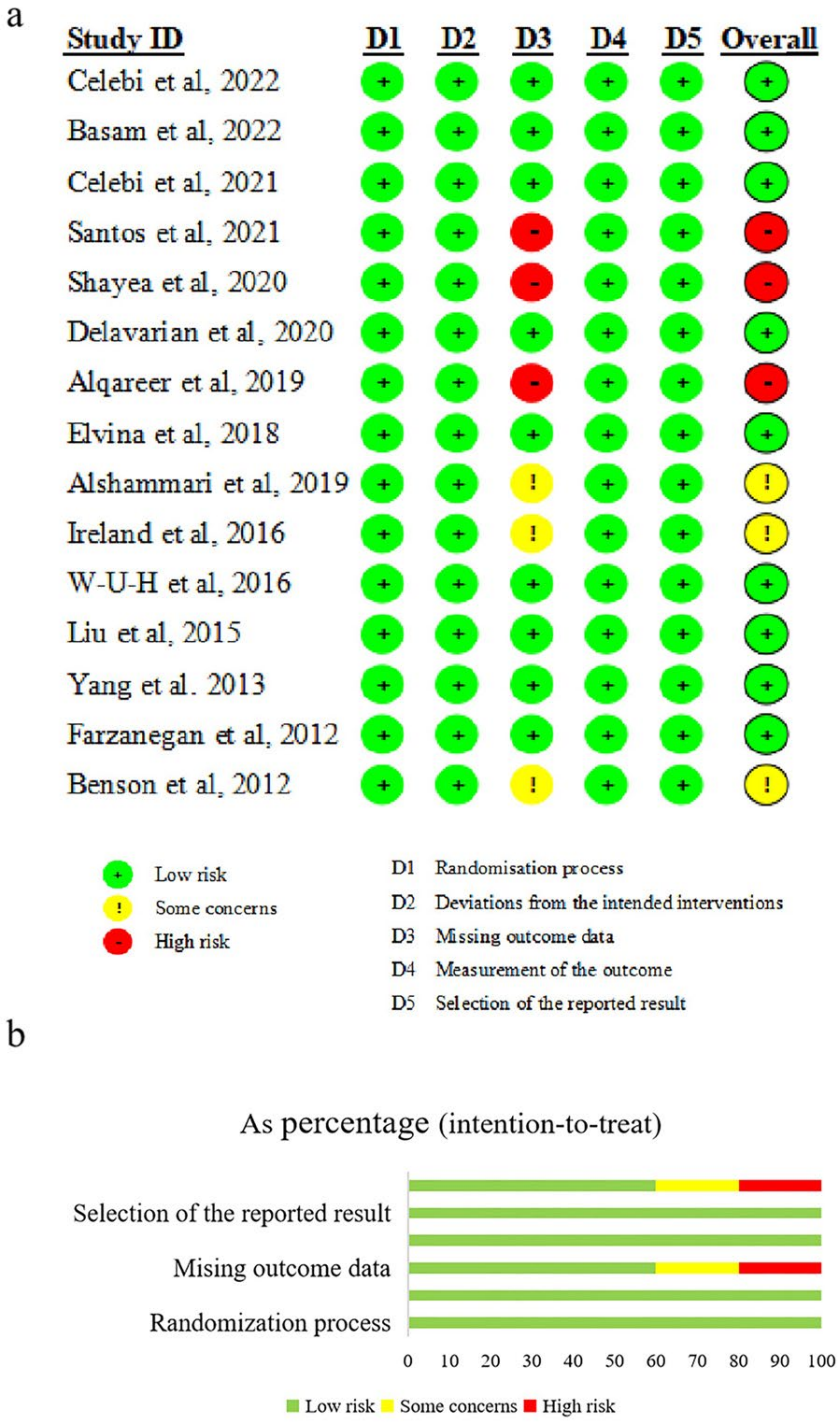
Risk of bias. **a** Risk of bias graph. **b** Risk of bias summary

Figure 2 illustrate the results of the risk of bias. According to the quality of the evidence, nine RCTs were low risk, three RCTs were high risk, and other RCTs was unclear risk. The higher risk was caused by the higher dropout due to some patients do not feel pain or taken analgesics.

### Data synthesis

**Fig. 3:**
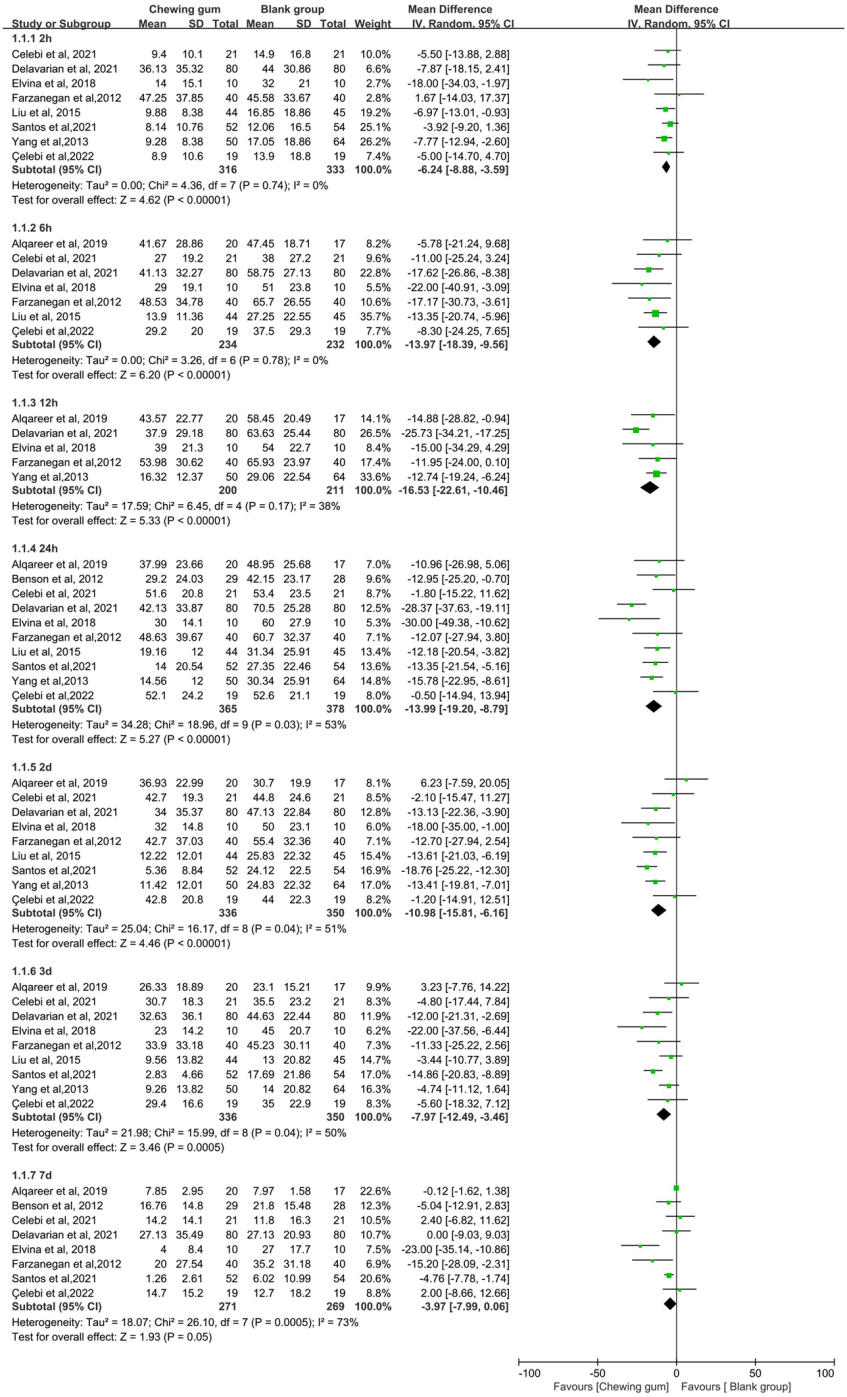
Forest plot of pain value between chewing gum group and blank group at different times

**Fig. 4:**
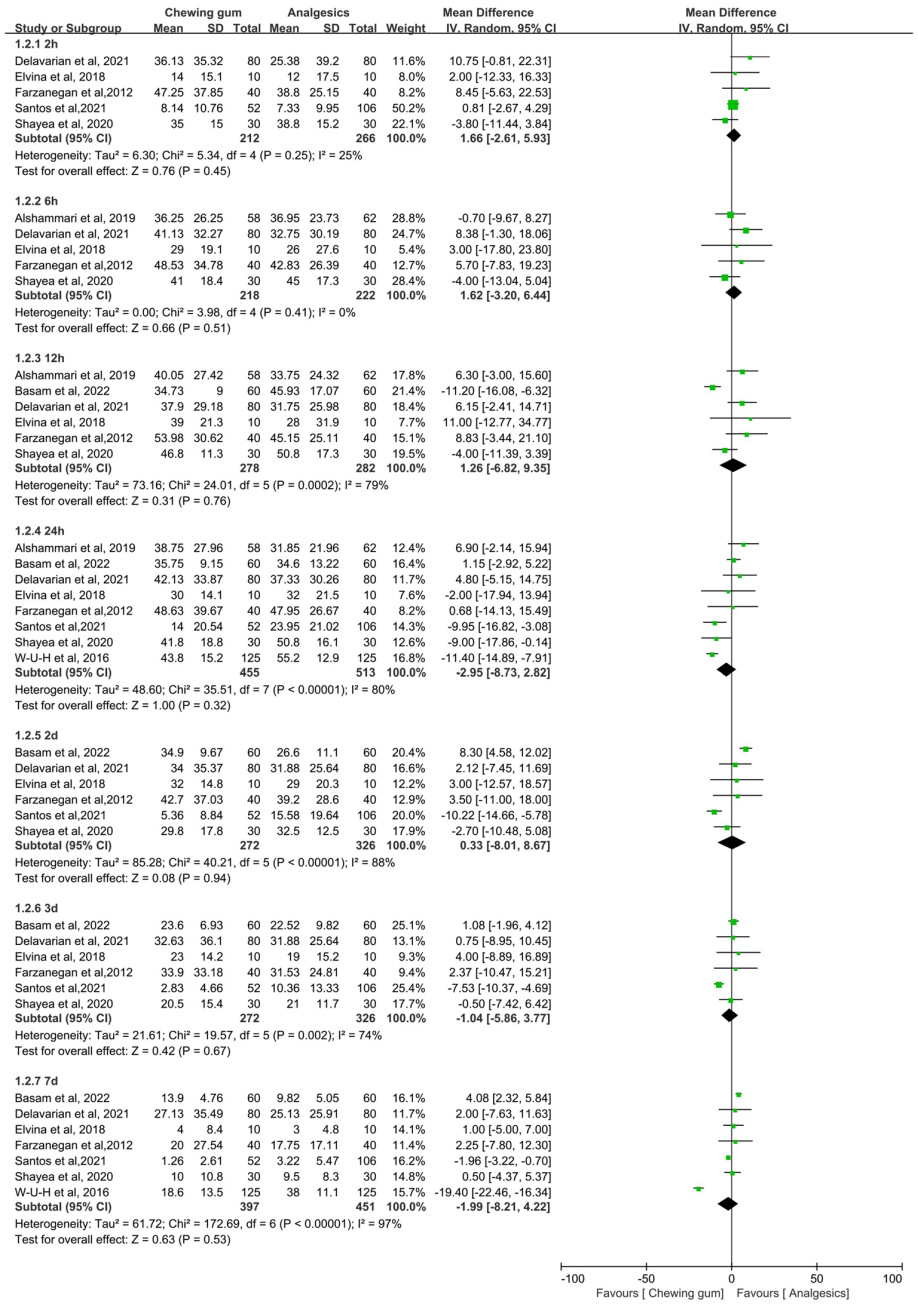
Forest plot of pain value between chewing gum group and analgesics group at different times

Ten studies evaluated pain scores after initial archwire placement in the chewing gum group and blank group [19–22, 24, 25, 29–31, 49]. As shown in Fig. 3, chewing gum showed a significant effect on pain relief for orthodontic patients compared to blank group at 2 h (MD = − 6.24, 95% CI − 8.88 to − 3.59, *P* < 0.00001, *I*^2^ = 0%), 6 h (MD = − 13.97, 95% CI − 18.39 to − 9.56, *P* < 0.00001, *I*^2^ = 0%), 12 h (MD = − 16.53, 95% CI − 22.61 to − 10.46, *P* < 0.00001, *I*^2^ = 38%), 24 h (MD = − 13.99, 95% CI − 19.20 to − 8.79, *P* < 0.00001, *I*^2^ = 53%), 2 days (MD = − 10.98, 95% CI − 15.81 to − 6.16, *P* < 0.00001, *I*^2^ = 51%), 3 days (MD = − 7.97, 95% CI − 12.49 to − 3.46, *P* = 0.0005, *I*^2^ = 50%) and 7 days (MD = − 3.97, 95% CI − 7.99 – 0.06, *P* = 0.05, *I*^2^ = 73%). Eight studies analysed orthodontic pain between the chewing gum group and the analgesics group [20, 22–28]. As shown in Fig. 4, no significant difference was found between the chewing gum group and the analgesics group at 2 h (MD = 1.66, 95% CI − 2.61–5.93, *P* = 0.45, *I*^2^ = 25%), 6 h (MD = 1.62, 95% CI − 3.20 – 6.44, *P* = 0.51, *I*^2^ = 0%),12 h (MD = 1.26, 95% CI − 6.82 – 9.35, *P* = 0.76, *I*^2^ = 79%), 24 h (MD = − 2.95, 95% CI − 8.73 − 2.82, *P* = 0.32, *I*^2^ = 80%), 2 days (MD = 0.33, 95% CI − 8.01–8.67, *P* = 0.94, *I*^2^ = 88%), 3 days (MD = − 1.04, 95% CI − 5.86–3.77, *P* = 0.67, *I*^2^ = 74%) and 7 days (MD = − 1.99, 95% CI − 8.21– 4.22, *P* = 0.53, *I*^2^ = 97%).

### Risk of bias across studies and additional analyses

**Fig. 5:**
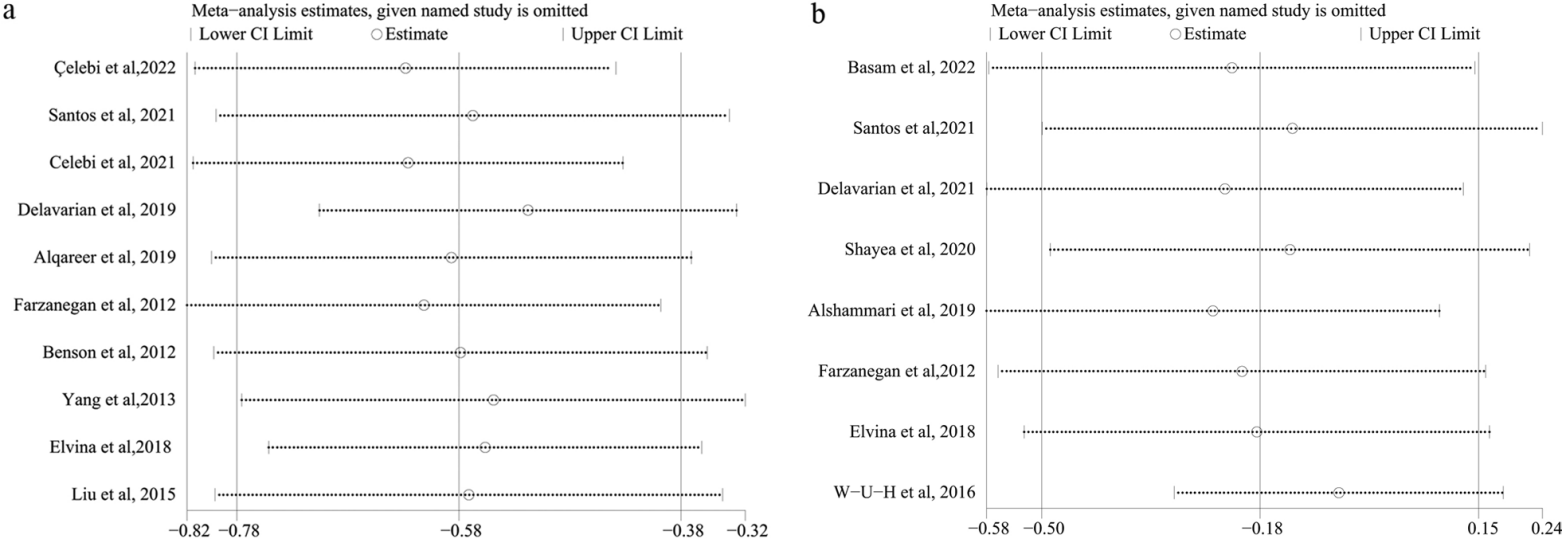
Results of sensitivity analysis. **a** sensitivity analysis for these studies compared the pain value between the gum group and the blank group; **b** sensitivity analysis for these studies compared the pain value between the gum group and the analgesics group

Pain is a subjective value, and clinical and demographic diversity existed across studies regarding participants' characteristics. Therefore, we chose the random effects model to estimate all pooled data. However, low heterogeneity was found at 2, 6, and 12 h, and moderate or severe heterogeneity at 24 h, 2 d, 3 d, and 7 d according to the I^2^ index when comparing the chewing gum group with the blank group (Fig. 3). The pooled results were not significantly different after excluding the included studies one by one (Fig. 5a). The Egger analysis showed no significant publication bias in included studies (*P* = 0.592 > 0.05). There was low heterogeneity at 2 h and 6 h, moderate heterogeneity at 3 d, and severe heterogeneity at 12 h, 24 h, 2 d, and 7 d when comparing chewing gum with analgesics (Fig. 4). The pooled results were not significant difference after removing the included studies one by one (Fig. 5b). The Egger analysis showed no significant publication bias in included studies (*P* = 0.489 > 0.05). The quality of the evidence across studies was evaluated according to the GRADE tool, and it was found that there was a very low quality of evidence (Table 2).

**Table 2:** Summary of overall quality of evidence of studies included in each meta-analysis using GRADE

Certainty assessment							
**Outcome**	**Number of Studies**	**Study Design**	**Risk of Bias**	**Inconsistency**	**Indirectness**	**Imprecision**	**Certainty**
Chewing gum group VS blank group	10	RCTs	Serious	Serious	Serious	Not serious	
Chewing gum group VS analgesics group	9	RCTs	Serious	Very serious	Serious	Serious	
GRADE Working Group grades of evidence							
High certainty: we are very confident that the true effect lies close to that of the estimate of the effect							
Moderate certainty: we are moderately confident in the effect estimate: the true effect is likely to be close to the estimate of the effect, but there is a possibility that it is substantially different							
Low certainty: our confidence in the effect estimate is limited: the true effect may be substantially different from the estimate of the effect							
Very low certainty: we have very little confidence in the effect estimate: the true effect is likely to be substantially different from the estimate of effect							

^a^Downgraded due to unclear or absence of blinding of both patients and outcome assessors

^b^Downgraded due to high heterogeneity

^c^Due to some Included studies that included only female subjects

^d^Downgraded due to credibility interval

## Discussion

Pain is considered the main negative aspect of orthodontic treatment, a deterrent to patient compliance, and the principal reason for discontinuation of treatment. Emerging studies have found that chewing gum, as a nonpharmacological method, has obvious effects on orthodontic pain relief [27, 28]. However, many orthodontists disagree with these views and believe that chewing gum will increase the rate of bracket loss, which is not conducive to the clinical application and promotion of chewing gum to relieve orthodontic pain. Therefore, this review is conducted to analyse the effect of chewing gum on relieving orthodontic pain compared to the blank group and analgesic group. In accordance with Mando et al.'s study [34], we found that chewing gum significantly reduced pain intensity when compared to the blank group. However, Mando and colleagues showed that chewing gum significantly reduced pain intensity compared to analgesics, which is inconsistent with our results. We found that the chewing gum had the same pain relief effect as analgesics, which was similar to Jabr et al.'s study [33]. This possibly because Mando et al.'s study included Ireland et al.'s multicenter RCTs in meta-analysis, although this study showed the differences between chewing gum and analgesic had no clinical importance in relieving orthodontic pain. In summary, we found that chewing gum significantly reduced orthodontic pain when compared to the blank group and had the equal pain relief effect when compared to analgesics. Nevertheless, chewing gum can be recommended as a suitable substitute for analgesics to reduce orthodontic pain.

Orthodontic pain is produced by metabolic activity in periodontal tissue caused by orthodontic force, including ischemia, inflammation, or edema in periodontal ligaments [50]. The mediators, such as prostaglandins, leukotrienes, histamine, substance P, bradykinin, dopamine, serotonin, glycine, glutamate gamma-aminobutyric acid, etc., released in periodontal tissue, initiate the inflammatory reaction, create the hyperalgesic response, and ultimately cause pain when orthodontic force is applied [3, 51, 52]. In addition, the pulp irritation caused by orthodontic tooth movement also induces orthodontal pain [53]. Therefore, it is believed that any factor that can temporarily displace the teeth under orthodontic force can resolve the pressure and prevent the formation of ischemic areas, thereby reducing pain.

Chewing gum has both local and central effects on pain relief [17]. Chewing gum increases blood flow into and around the periodontal membrane, loosens tightly grouped fibers around nerves and blood vessels, restores normal vascular and lymphatic circulation, and prevents or relieves inflammation in the periodontal tissue, thereby reducing pain [50]. Meanwhile, chewing gum for 20 min activated the ventral part of the prefrontal cortex and evoked augmented activity of 5-HT neurons in the dorsal raphe nucleus and, therefore, suppressed nociceptive responses [54]. Chewing gum also has pharmacological pain-relieving effects for orthodontic pain [55]. Distraction is an effective way to reduce pain, because the brain can only focus on one thing at a time. Chewing gum can transfer patients' attention to mastication, reduce the neuronal response to the harmful stimulus, and make them feel happy. Sometimes, patients can even release pain or irritability by chewing gum.

Chewing gum has great benefits in relieving pain compared to analgesics. It has the advantages of noninvasive, inexpensive and convenient, and avoids the side effects caused by analgesics. Simultaneously, chewing gum has other benefits. Chewing gum can be a simple and effective way to reduce stress and tension, and it can enhance α brain wave, which is a sign that the spirit is in a calm state. Chewing gum is beneficial to improve digestive function by stimulating saliva secretion to promote swallowing and digestive activity. Chewing gum is also beneficial for oral cleaning and reduces the occurrence of demineralization and caries by increasing the saliva flow rate and PH value [56–58]. In addition, gum can be used as a carrier for drugs or active molecules to improve its function. For example, chewing gum containing sodium metaphosphate can effectively remove coffee stains [59], chewing gum containing potassium chloride can reduce dental hypersensitivity for a long time [60], and chewing gum containing analgesics can enhance its pain relief effect [61].

In addition, bracket breakage is one of the factors affecting patient satisfaction, and many doctors believe that chewing gum will cause bracket breakage, which will not only increase the time of operation beside the chair but also prolong the treatment cycle. Four studies evaluated the effect of chewing gum on the rate of appliance breakage and found that chewing gum did not increase the rate of bracket breakage when compared to the blank group or analgesics group [23, 27, 48, 49]. Moreover, chewing gum will not increase the rate of bracket breakages but will be beneficial to oral health and dental caries [56].

There was moderate or severe heterogeneity in some pooled results according to the I^2^, which were caused by clinical heterogeneity, methodological heterogeneity and statistical heterogeneity in this meta-analysis. Although all studies are well-designed RCTs, it is inevitable that there are some differences in participants characteristics, malocclusion, types of fixed appliances, treatment plan, clinical operation, etc. In addition, pain is a subjective feeling that is affected by many factors, such as age, gender, pain threshold, cultural differences, etc. Therefore, we applied the random effect model and performed sensitivity analysis. The sensitivity analysis showed no significant difference for the pooled results after excluding the included studies one by one.

Although this meta-analysis was conducted carefully, there were still some limitations. Orthodontic pain is a subjective feeling that is influenced by psychological, physiological, social and other factors, such as the patient's age [62], gender [63], type of orthodontic appliances [64, 65], magnitude of orthodontic force [66], treatment motivation [66], expectations of orthodontic treatment outcomes [67], emotional state [68] and personality traits [69]. Similarly, patient characteristics, including the crowding degree of dentition, whether tooth extraction or not, single or two dental arches, and the size of the initial arch wire, will affect the patient's pain intensity. Although each study noted that they included similar participants, the baseline characteristics of participants in each study were not always the same. In addition, the level of certainty of the meta-analysis results was assessed as very low level of certainty according GRADE tool. Therefore, more well-designed RCT studies with large samples are needed to obtain more reliable conclusions in the future.

## Conclusions

This review has demonstrated that chewing gum has a significant effect on relieving orthodontic pain and can be recommended as a safe, low-cost and convenient alternative to analgesics with no side effects to reduce orthodontic pain during fixed orthodontic appliances in daily orthodontic practice.

## Data Availability

The data sets used and/or analysed during the current study are available from the corresponding author on reasonable request.

## Abbreviations

PRISMA: Preferred Reporting Items for Systematic Review and Meta-Analysis
RCT: Randomized clinical trial
VAS: Visual–analog scale
NSAIDs: Nonsteroidal anti-inflammatory drugs
LLLT: Low-level laser therapy
TENS: Transcutaneous electrical nerve stimulation
LIPUS: Low-intensity pulsed ultrasound
NRS: Numeric Rating Scale
